# Minimal Clinically Important Differences for the Modified Rodnan Skin Score: Results from the Scleroderma Lung Studies (SLS-I and SLS-II)

**DOI:** 10.1186/s13075-019-1809-y

**Published:** 2019-01-16

**Authors:** Dinesh Khanna, Philip J. Clements, Elizabeth R. Volkmann, Holly Wilhalme, Chi-hong Tseng, Daniel E. Furst, Michael D. Roth, Oliver Distler, Donald P. Tashkin

**Affiliations:** 1 Department of Medicine, University of Michigan, Ann Arbor, MI 48105 USA; 2 Department of Medicine, David Geffen School of Medicine at University of California, Los Angeles, CA USA; 3 Department of Rheumatology, University Hospital Zurich, Zurich, Switzerland; 4 Division of Rheumatology, Department of Internal Medicine, University of Michigan Scleroderma Program, 300 North Ingalls Street, SPC 5422, Ann Arbor, MI 48109 USA

**Keywords:** Interstitial lung disease, Systemic sclerosis, Minimal clinically important difference, Skin thickness, Scleroderma, Modified Rodnan skin score

## Abstract

**Objective:**

This study aimed to assess the minimal clinically important differences (MCIDs) for the modified Rodnan skin score (mRSS) using combined data from the Scleroderma Lung Studies (I and II).

**Methods:**

MCID estimates for the mRSS at 12 months were calculated using three anchors: change in scores on the Health Assessment Questionnaire- Disability Index from baseline to 12 months, change in scores on the Patient Global Assessment from baseline to 12 months, and answer at 12 month for the Short Form-36 health transition question “Compared to one year ago, how would you rate your health in general now?” We determined the mRSS MCID estimates for all participants and for those with diffuse cutaneous systemic sclerosis (dcSSc). We then assessed associations between MCID estimates of mRSS improvement and patient-reported outcomes, using Student’s *t* test to compare the mean differences in patient outcomes between those who met the MCID improvement criteria versus those who did not meet the improvement criteria.

**Results:**

The mean (SD) mRSS at baseline was 14.75 (10.72) for all participants and 20.93 (9.61) for those with dcSSc. The MCID estimate for mRSS improvement at 12 months ranged from 3 to 4 units for the overall group (improvement of 20–27% from baseline) and was 5 units for those with dcSSc (improvement of 24% from baseline). Those who met the mRSS MCID improvement criteria had statistically significant improvements in scores on the Short Form-36 Physical Component Summary, the Transition Dyspnea Index, and joint contractures at 12 months.

**Conclusion:**

MCID estimates for the mRSS were 3–4 units for all participants and 5 units for those with dcSSc. These findings are consistent with previously reported MCID estimates for systemic sclerosis.

## Introduction

Systemic sclerosis (SSc; scleroderma) is a multiorgan disease with a complex interplay of diverse pathological processes involving inflammation, fibrosis, and vasculopathy. While organ involvement in SSc varies, skin involvement is almost universal in SSc [1]. The modified Rodnan skin score (mRSS), a measure of skin thickness, is the primary outcome measure in the majority of clinical trials of diffuse cutaneous SSc (dcSSc). Measurement of skin thickness is used as surrogate measure of disease severity and mortality in patients with dcSSc. Specifically, an increase in skin thickening is associated with involvement of internal organs and increased mortality [1]. It is generally accepted that the mRSS tends to worsen in the early part of the disease and to improve in late disease, although the time of peak involvement is poorly defined. The mRSS is feasible, reliable, valid, and sensitive to change in multicenter clinical trials [2].

To interpret change in the mRSS within a group of participants with SSc over time, or to interpret differences in the mRSS between two groups, it is important to first define whether the change or difference is clinically meaningful. The minimal clinically important difference (MCID) is defined as the smallest difference in a measure or instrument that is considered to be “worthwhile or important” to patients [3]. For clinicians, the MCID helps guide treatment. While there are several methods for calculating the MCID, estimating the MCID using an external anchor is often preferred over other methods [4]. Khanna et al. [5] previously published MCID estimates for the mRSS using data from the d-penicillamine trial, which involved a cohort of patients with early dcSSc. They found that an improvement of 3.2–5.3 units for the mRSS were the MCID estimates. However, they used physician assessment of change over time as the anchor to determine the MCID estimates; it is preferred that this information comes directly from patients [4]. Thus, in this article, we analyzed data from two clinical trials in SSc-related interstitial lung disease—the Scleroderma Lung Studies I and II (SLS-I and SLS-II)—to calculate the MCID estimates for the mRSS using patient-reported anchors from participants with SSc.

## Methods

### Participants

All participants with any outcome data in SLS-I and SLS-II were evaluated in this post-hoc analysis. The study protocols for both SLS-I and SLS-II were approved by the local Institutional Review Boards, and written informed consent was obtained from all participants. The trial designs for both SLS-I and SLS-II have been published elsewhere [6, 7]. Briefly, participants meeting the 1980 SSc classification criteria were included. Participants in SLS-I were randomized to 1 year of oral placebo or oral cyclophosphamide, with the primary endpoint being change in FVC percent predicted (FVC%) at 1 year. Participants in SLS-II were randomized to 2 years of mycophenolate mofetil or 1 year of oral cyclophosphamide followed by 1 year of placebo. The primary endpoint for SLS-II was the course of the FVC% from baseline to 24 months using a joint model, which examined the repeated measurements of FVC%. The mRSS was captured as a secondary outcome measure in both trials [8] and was assessed by experienced rheumatologists at Scleroderma Centers for Excellence throughout the trials. We did not perform mRSS training sessions before each trial.

### Methods and procedures

Participants’ clinical data included age, gender, race, disease duration (from first non-Raynaud’s symptom attributable to SSc), skin subtype of SSc (dcSSc or limited cutaneous SSc (lcSSc)), presence of tendon friction rubs (captured as yes or no at baseline and month 12), presence of small and large joint contractures (captured as yes or no at baseline and month 12), and the mRSS. In both studies, the patient-reported outcome measures included the Mahler Baseline and Transition Dyspnea Indexes (BDI and TDI), the Health Assessment Questionnaire (HAQ-DI), the Medical Outcomes Short Form-36 (SF-36), and the Patient Global Assessment of Disease Activity (PtGA) [9].

The *Mahler Baseline and Transition Dyspnea Indexes* (BDI and TDI) [10] measured patient dyspnea. The BDI measured patient dyspnea at baseline, and the TDI measured their change from baseline to 12 months. Scores ranged from − 3 to + 3 for three domains, for a sum ranging between − 9 and + 9. Higher positive scores connoted less dyspnea (BDI) or an improvement in dyspnea (TDI). While both the BDI and the TDI were assessed using paper questionnaires in SLS-I, both indices were assessed using a self-administered, computer-generated format in SLS-II.

The *Health Assessment Questionnaire* (HAQ-DI) is a 20-item questionnaire assessing patients’ functional disability in eight domains. Scores ranged from 0.0 (best) to 3.0 (worst) [11]. It has been fully validated in SSc [12].

The *Medical Outcomes Short Form-36* (SF-36) version 2 is a self-administered survey assessing patients’ generic health-related quality of life. It generated a physical component summary and a mental component summary [13] and was scored on a *t*-score metric with a US population mean of 50 (SD 10); a higher score denoted better health-related quality of life. One item is a health transition question and asked the patient whether their health had got better or worse, as described in more detail in the following. The SF-36 has been previously validated in SSc [14].

### Statistical analysis

Summary statistics were calculated for all demographic and clinical variables. Continuous variables are reported as mean and standard deviation (SD), and frequencies are reported for categorical variables.

#### Anchors to assess the MCID

To determine the MCID, we used three anchors directly from participants: the health transition question from the SF-36 answered at 12 months, the change in their HAQ-DI score from baseline to 12 months, and the change in their PtGA score from baseline to 12 month visit. We chose these anchors based on their relationships to the mRSS in previous studies [15] and because all of this information is provided directly by the patient. Additionally, experts recommend multiple anchors to get robust estimates [4]. The SF-36 health transition question asks the patient: “Compared to one year ago, how would you rate your health in general now?” We used the “somewhat better” and “somewhat worse” responses as the anchors for calculating the MCID [16–18]. For the HAQ-DI, we chose a previously published MCID estimate of 0.14 units for improvement in SSc [5] and other arthritides [0.22], and we used an arbitrary cutoff point of 0.48 because large improvements in the HAQ-DI score may be greater than the MCID. For the PtGA, we used a cutoff point of 20 units (0–100 units) as the MCID, and we used an arbitrary cutoff point of 50 units as a change that is greater than the MCID.

We assessed the appropriateness of the anchors by calculating Spearman correlations between the anchors (changes in HAQ-DI scores, changes in PtGA scores, and the SF-36 health transition answer at 12 months) and changes in the mRSS from baseline to 12 months. Generally, a correlation coefficient of ≥ 0.30 is considered acceptable [4, 19]. If we did not achieve this threshold, we considered *p* < 0.05 as an acceptable alternative. We also sought to determine whether the MCID estimates for changes in the mRSS were associated with changes in several patient-reported outcomes (i.e., the SF-36 Physical and Mental Component Summary Scores and the TDI), and changes in physical examination (improvement in tendon friction rubs and joint contractures) due to their relationship with mRSS [20].

Student’s *t* tests or chi-square tests were used to compare the mean difference in patient-reported outcomes or in percent difference between those who had improved mRSS as defined by the MCID estimates versus those whose mRSS scores did not improve as defined by the MCID. We calculated the effect size as the mean change in the mRSS divided by the SD at baseline. *p* < 0.05 was considered statistically significant and no adjustment was made for multiple testing.

## Results

### Baseline characteristics and changes in mRSS and patient-reported outcomes

We evaluated data from 300 participants in SLS-I and SLS-II combined (158 participants in SLS-I and 142 participants in SLS-II; Table 1). The mean (SD) age of the pooled cohort was 50.3 (11.3) years, mean (SD) disease duration was 2.9 (2.0) years, mean (SD) FVC% at baseline was 67.4% (10.8%), and 59% of the participants had dcSSc in both trials (Table 1). While both studies were comparable in most baseline characteristics, those in SLS-II were older (*p* = 0.004), had shorter mean disease duration (*p* = 0.01), and had less baseline dyspnea as assessed by the BDI (data not shown; 7.2 vs 5.7, *p* < 0.001).

**Table 1:** Baseline demographics for all participants

Variable	Category	Overall		Diffuse SSc		Limited SSc		*p* value
		*N*	Mean (SD)	*N*	Mean (SD)	*N*	Mean (SD)	
Age, mean (SD) (years)		300	50.3 (11.3)	177	49.3 (11.5)	123	51.6 (10.9)	0.08
Disease duration (years)		297	2.9 (2.0)	176	2.9 (1.9)	121	2.9 (2.1)	0.79
Gender, *N* (%)	Male		84 (28%)		54 (31%)		30 (24%)	0.25
	Female		216 (72%)		123 (69%)		93 (76%)	
SSc type, *N* (%)	Diffuse		177 (59%)		177 (100%)		0 (0%)	
	Limited		123 (41%)		0 (0%)		123 (100%)	
Race, *N* (%)	Caucasian		177 (59%)		101 (57%)		76 (62%)	0.12
	Black		58 (19%)		41 (23%)		17 (14%)	
	Other		65 (22%)		35 (20%)		30 (24%)	
mRSS—baseline, mean (SD)	Overall	300	14.75 (10.72)	177	20.93 (9.61)	123	5.85 (3.61)	< 0.0001
mRSS—12 months, mean (SD)		244	11.90 (9.91)	146	16.10 (10.41)	98	5.64 (4.17)	< 0.0001
Patient Global Assessment—baseline, mean (SD)		249	39.0 (26.9)	145	43.5 (26.6)	104	32.7 (26.1)	0.002
HAQ-DI--baseline, mean (SD)		299	0.78 (0.67)	177	0.99 (0.72)	122	0.48 (0.49)	< 0.0001
BDI,--baseline mean (SD)		287	6.4 (2.2)	171	6.4 (2.2)	116	6.4 (2.2)	0.97
SF-36 PCS--baseline, mean (SD)		285	34.7 (10.5)	169	32.9 (10.6)	116	37.4 (9.8)	< 0.0001
SF-36 MC--baselineS, mean (SD)		285	49.5 (9.9)	169	48.7 (10.0)	116	50.6 (9.5)	0.11
Joint contractures present at baseline, *N* (%)			143 (48%)		111 (63%)		32 (26%)	< 0.0001
Tendon friction rubs present at baseline, *N* (%)			36 (13%)		34 (20%)		2 (2%)	< 0.0001

*BDI* Baseline Dyspnea Index, *HAQ-DI* Health Assessment Questionnaire Disability Index, *mRSS* modified Rodnan Skin Score, *SD* standard deviation, *SF-36 MCS* Short Form-36 Mental Component Summary, *SF-36 PCS* Short Form-36 Physical Component Summary, *SSc* systemic sclerosis

The mean (SD) mRSS at baseline for all participants was 14.75 (10.72), but the mRSS was higher for those with dcSSc and lower for those with lcSSc as expected (Table 1). The change in mRSS was − 2.84 (5.91) in the overall group, − 4.49 (6.75) in dcSSc participants, and − 0.37 (3.00) in lcSSc participants.

### Correlation coefficients to assess the appropriateness of MCID anchors

For our MCID analysis, the correlation coefficients between the anchors (SF-36 health transition at 12  month period and changes in HAQ-DI and PtGA from baseline to 12 months) and the change in the mRSS from baseline to 12 months did not meet the 0.30 threshold (Table 2). However, the correlations for the overall group were statistically significant, thus meeting our alternative criteria and indicating that the anchors are acceptable. To better understand this, we calculated the correlations separately for those participants with dcSSc and those with lcSSc. For those participants with dcSSc, the coefficients were also below the 0.30 threshold, but all three correlation coefficients were statistically significant. For those participants with lcSSc, the coefficients were very small and not statistically significant.

**Table 2:** Spearman correlation coefficients between change in mRSS and the three patient-reported anchors

	Overall	Diffuse SSc	Limited SSc
Health transition	0.19 (*p* = 0.007)	0.21 (*p* = 0.02)	− 0.03 (*p* = 0.81)
	*n* = 187	*n* = 115	*n* = 72
PtGA	0.21 (*p* = 0.004)	0.20 (*p* = 0.02)	0.11 (*p* = 0.33)
	*n* = 195	*n* = 118	*n* = 77
HAQ-DI	0.24 (*p* = 0.0002)	0.26 (*p* = 0.001)	0.10 (*p* = 0.34)
	*n* = 243	*n* = 146	*n* = 97

*HAQ-DI* Health Assessment Questionnaire Disability Index, *mRSS* modified Rodnan Skin Score, *PtGA* Patient Global Assessment, *SSc* systemic sclerosis

### mRSS MCID estimates for reported improvement in the overall group

We provide unadjusted mRSS MCID estimates for both improvement and no change in all three anchors (Tables 3, 4, and 5). For the SF-36 health transition question, the mean mRSS MCID estimate for reported improvement (defined as answering “somewhat improved”) was − 2.86 with an effect size of 0.27. This estimate was similar to the group that reported no change for the question, which had an estimate of 2.72 with an effect size of 0.25 (Table 3). Using the HAQ-DI cutoff points described earlier, the mRSS MCID estimate for HAQ-DI improvement was − 3.56 with an effect size of 0.33, and this was numerically larger than the HAQ-DI no change group (mean change of − 2.55 and effect size of 0.24; Table 4). Using the PtGA cutoff points described earlier, the mRSS MCID estimate for PtGA improvement was − 4.06 with an effect size of 0.38, and this was numerically larger than the PtGA no change group (mean change of − 2.94 and effect size of 0.27; Table 5).

**Table 3:** Estimation of MCID estimates in mRSS using SF-36 health transition anchor for all participants

Change in mRSS by level of anchors—SF-36 health transition item	*N*	Unadjusted mean			Effect size
		Mean	95% LCL	95% UCL	
Overall					
Much better (1)	50	− 5.64	− 7.31	− 3.97	− 0.53
Somewhat better (2) (MCID estimate for improvement)	35	− 2.86	− 4.94	− 0.78	− 0.27
Same (3)	67	− 2.72	− 3.96	− 1.48	− 0.25
Somewhat worse (4)	32	− 1.66	− 4.04	0.73	− 0.15
Much worse (5)	3	− 4.67	− 20.64	11.3	− 0.44
Diffuse SSc					
Much better (1)	35	− 7.66	− 9.64	− 5.67	− 0.80
Somewhat better (2) (MCID estimate for improvement)	23	− 4.70	− 7.38	− 2.01	− 0.49
Same (3)	36	− 4.61	− 6.56	− 2.66	− 0.48
Somewhat worse (4)	20	− 2.25	− 6.12	1.62	− 0.23
Much worse (5)	1	− 12.00			
Limited SSc					
Much better (1)	15	− 0.93	− 2.28	0.41	− 0.26
Somewhat better (2) (MCID estimate for improvement)	12	0.67	− 1.82	3.15	0.19
Same (3)	31	− 0.52	− 1.61	0.58	− 0.14
Somewhat worse (4)	12	− 0.67	− 1.76	0.43	− 0.19
Much worse (5)	2	− 1.00	− 13.71	11.71	− 0.28

*LCL* lower confidence limit, *MCID* minimal clinically important difference, *mRSS* modified Rodnan Skin Score, *SF-36* Short Form-36, *SSc* systemic sclerosis, *UCL* upper confidence limit

**Table 4:** Estimation of MCID estimates in mRSS using HAQ-DI anchor for all participants

HAQ-DI	*N*	Unadjusted mean			Effect size
		Mean	95% LCL	95% UCL	
Overall					
Change ≤ − 0.48	38	− 6.11	− 8.29	− 3.92	− 0.57
− 0.14 ≥ change > − 0.48 (MCID estimate for improvement)	34	− 3.56	− 6	− 1.12	− 0.33
− 0.14 < change < 0.14	108	− 2.55	− 3.47	− 1.62	− 0.24
0.14 ≤ change < 0.48	26	− 0.88	− 2.84	1.07	− 0.08
Change ≥ 0.48	37	− 1.03	− 3.14	1.09	− 0.10
Diffuse SSc					
Change ≤ − 0.48	29	− 7.97	− 10.41	− 5.52	− 0.83
− 0.14 ≥ change > − 0.48 (MCID estimate for improvement)	22	− 4.64	− 8.28	− 0.99	− 0.48
− 0.14 < change < 0.14	54	− 4.8	− 6.23	− 3.36	− 0.50
0.14 ≤ change < 0.48	16	− 1.69	− 4.88	1.5	− 0.18
Change ≥ 0.48	25	− 1.48	− 4.56	1.6	− 0.15
Limited SSc					
Change ≤ − 0.48	9	− 0.11	− 2.3	2.08	− 0.03
− 0.14 ≥ change > − 0.48 (MCID estimate for improvement)	12	− 1.58	− 3.74	0.57	− 0.44
− 0.14 < change < 0.14	54	− 0.3	− 1.15	0.56	− 0.08
0.14 ≤ change < 0.48	10	0.4	− 0.68	1.48	0.11
Change ≥ 0.48	12	− 0.08	− 1.99	1.82	− 0.02

*HAQ-DI* Health Assessment Questionnaire -Disability Index, *LCL* lower confidence limit, *MCID* minimal clinically important difference, *mRSS* modified Rodnan Skin Score, *SSc* systemic sclerosis, *UCL* upper confidence limit

**Table 5:** Estimation of MCID estimates in mRSS using Patient Global Assessment anchor for all participants

Patient Global Assessment	*N*	Unadjusted mean			Effect size
		Mean	95% LCL	95% UCL	
Overall					
Change ≤ − 50	8	− 3.75	− 7.69	0.19	− 0.35
−20 ≥ change > − 50 (MCID estimate for improvement)	35	− 4.06	− 6.66	− 1.45	− 0.38
− 20 < change < 20	123	− 2.94	− 3.92	− 1.96	− 0.27
20 ≤ change < 50	23	− 0.17	− 2.71	2.36	− 0.02
Change ≥ 50	6	1.5	− 2.94	5.94	0.14
Diffuse SSc					
Change ≤ − 50	5	− 5.60	− 11.85	0.65	− 0.58
−20 ≥ change > − 50 (MCID estimate for improvement)	26	− 5.12	− 8.48	− 1.75	− 0.53
− 20 < change < 20	69	− 5.16	− 6.57	− 3.74	− 0.54
20 ≤ change < 50	16	− 0.56	− 4.25	3.12	− 0.06
Change ≥ 50	2	6.50	− 25.27	38.27	0.68
Limited SSc					
Change ≤ − 50	3	− 0.67	− 5.84	4.50	− 0.19
− 20 ≥ change > − 50 (MCID estimate for improvement)	9	− 1.00	− 3.72	1.72	− 0.28
− 20 < change < 20	54	− 0.11	− 0.98	0.76	− 0.03
20 ≤ change < 50	7	0.71	− 1.40	2.83	0.20
Change ≥ 50	4	− 1.00	− 2.30	0.30	− 0.28

*LCL* lower confidence limit, *MCID* minimal clinically important difference, *mRSS* modified Rodnan Skin Score, *SSc* systemic sclerosis, *UCL* upper confidence limit

### mRSS MCID estimates for reported improvement in those with dcSSc

For those participants with dcSSc, using the SF-36 health transition question, the mean mRSS MCID estimate for those who reported improvement was − 4.70 with an effect size of 0.49. This was similar to the group reporting no change, which had an estimate of − 4.61 with an effect size of 0.48 (Table 3). Using the HAQ-DI, the mean mRSS MCID estimate for HAQ-DI improvement was − 4.64 with an effect size of 0.48, and this was similar to the group with no change in HAQ-DI (mean change of − 4.8 and effect size of 0.50; Table 4). Using the PtGA, the mean mRSS MCID estimate for PtGA improvement was − 5.12 with an effect size of 0.53, and this was similar to the group with no change in PtGA (mean change of − 5.16 and effect size of 0.54; Table 5).

### mRSS MCID estimates for reported improvement in those with lcSSc

For those participants with lcSSc, the mRSS MCID estimates were small and within the measurement error of the mRSS (Tables 3, 4, and 5). Specifically, the mRSS MCID estimate was 0.67 for improvement on the SF-36 health transition question, − 1.58 for improvement in HAQ-DI scores, and − 1.0 for improvement in PtGA scores.

### mRSS MCID estimates for reported worsening in the cohort

On average, the mRSS improved in participants who categorized themselves as “somewhat worse” on health transition, and using definitions of worsening in HAQ-DI and PtGA (Table 3, 4, and 5). These trends were also seen in those specifically with dcSSc.

### Relationship between the mRSS MCID estimates, patient-reported outcomes, and musculoskeletal examination

We explored whether the participants whose mRSS scores improved by ≥ MCID over 12 months had parallel changes in patient-reported outcome scores (Table 6). We used an improvement of > 3 and 4 units of the mRSS for the overall group and > 5 units of the mRSS for those with dcSSc. For the participants who met these mRSS MCID improvement criteria, they also had significantly greater improvements for the SF-36 physical component summary and the TDI compared to those whose mRSS changes did not meet the MCID estimates (*p* < 0.05 for all comparisons), for both the overall group and for those with dcSSc. For those with dcSSc, a greater proportion of participants who met the mRSS MCID criteria had an improvement in their small and large joint contractures compared to those who did not meet the MCID improvement criteria (*p* = 0.008).

**Table 6:** Change in patient-reported outcome measures and musculoskeletal involvement by the MCID estimates for all participants and for diffuse cutaneous SSc

	Overall *N*	SF-36 PCS			SF-36 MCS			TDI			Joint contractures^b^			Tendon friction rubs^b^		
		*N*	Mean difference	*p* value^a^	*N*	Mean difference	*p* value^a^	*N*	Mean difference	*p* value^a^	*N*	*N* (% improved)	*p* value	*N*	*N* (% improved)	*p* value
Change in mRSS > 5 improvement in diffuse cutaneous SSc																
12 months																
Change ≥ − 5 (no change/worsening group)	82	62	0.59 (8.35)	Ref.	62	1.79 (11.52)	Ref.	76	0.32 (2.90)	Ref.	53	2 (4%)	0.01	12	9 (75%)	0.99
Change <− 5 (improvement group)	64	54	3.62 (8.20)	0.05	54	3.05 (9.03)	0.52	59	1.85 (4.03)	0.01	40	9 (23%)		12	9 (75%)	
Change in mRSS > 3 improvement in all participants																
12 months																
Change ≥ − 3 (no change/worsening group)	143	102	−0.01 (8.59)	Ref.	102	0.90 (11.67)	Ref.	132	0.04 (3.53)	Ref.	61	7 (12%)	0.3	8	5 (63%)	0.33
Change <− 3 (improvement group)	101	101	2.66 (8.11)	0.03	85	3.27 (8.84)	0.12	92	1.34 (3.72)	0.01	56	10 (18%)		18	15 (83%)	
Change in mRSS > 4 improvement in all participants																
12 months																
Change ≥ − 4 (no change/worsening group)	159	115	0.05 (8.41)	Ref.	115	1.37 (11.36)	Ref.	144	0.03 (3.41)	Ref.	68	7 (10%)	0.1	12	9 (75%)	0.99
Change <− 4 (improvement group)	85	72	3.06 (8.26)	0.02	72	2.93 (9.00)	0.33	80	1.55 (3.90)	0.01	49	10 (20%)		14	11 (79%)	

*MCID* minimal clinically important difference, *mRSS* modified Rodnan Skin Score, *Ref.* reference, *SF-36 MCS* Short Form-36 Mental Component Summary, *SF-36 PCS* Short Form-36 Physical Component Summary, *SSc* systemic sclerosis, *TDI* Transition Dyspnea Index

^a^*t* test of whether change is significantly different from “no change” group

^b^Analysis set for joint contractures and tendon friction rubs includes only participants with present joint contractures (small or large) or tendon friction rubs at baseline. Only participants with data at both baseline and 12 months are included. Chi-square tests (or Fisher’s exact tests where appropriate) were used to compare percentage of participants who improved by MCID group

## Discussion

The mRSS is a surrogate of skin and internal organ involvement in SSc and is feasible, reliable, valid, and sensitive to change in multicenter clinical trials [2]. Since the mRSS serves as the primary outcome measure in recent clinical trials, it is important to define which changes in the mRSS between two groups are clinically meaningful (not just statistically significant). Additionally, for the clinician, the MCID provides information on treatment response and can help guide treatment. Our data suggest that an improvement of 3–4 units in all SSc patients (an improvement of 20–27% from the baseline score) and an improvement of 5 units in dcSSc patients (an improvement of 24% from baseline scores) are appropriate MCID estimates.

Our data align with the mRSS MCID estimates from the dPenicillamine trial (based on physician report), where an improvement of 3.5–5.3 units was considered the MCID [5]. Another study including the physician consensus exercise also defined an improvement of 3.0–7.5 units as the mRSS MCID estimates for improvement [21]. Additionally, improvements of > 5 units and ≥ 25% have recently been considered clinically important estimates for the mRSS in dcSSc [22]. Thus, our findings are very similar to published estimates. In addition, our MCID estimates are associated with statistically significant changes in the SF-36 physical component summary and TDI as well as improvement in small and large joint contractures, suggesting that these MCID estimates translate into *how a patient feels and functions* [23].

MCID estimates are calculated at a group level and should not be confused with change in a measure in an individual patient. At an individual level, a larger change is required to be considered a statistically significant change, and it is influenced by both measurement error and normal biologic variability over time [4]. It may also be influenced by the severity of disease and skin involvement.

Our data highlight a few important things about assessing the MCID estimates. First, despite the large number of participants in the two trials, the lack of appropriate correlations between the anchors and the mRSS indicate uncertainty in the point estimates. This is highlighted by the similar mean changes in the mRSS for both the MCID improved and no change groups for those with dcSSc. Second, using the patient anchors of SF-36 health transition, HAQ-DI, and PtGA, patients who worsened on these anchors still had, on average, an overall improvement in the mRSS (with wide CIs that crossed 1). Although this seems surprising, this is likely due to the poor correlation coefficients between the anchors and the mRSS, as the SF-36 health transition question and the PtGA survey assess overall change in health (beyond improvement in skin) and the HAQ-DI asks about daily functional activities [15]. In previous analyses from a dcSSc cohort, the mRSS had a larger correlation with the physician global assessment compared to the PtGA [15]. Incorporating anchors that focus on change in skin involvement and its impact on function and other daily activities may be more appropriate and should be considered in future trials. Third, as expected, those with lcSSc had minimal change in the mRSS during the two SSc trials. In our analyses, the change in the mRSS for those with lcSSc was ≤ 1.0 units in a majority of the subgroups.

Our study has many strengths. We used prospective data from two large SSc randomized controlled trials (SLS-I and SLS-II) to determine MCID estimates. Although the mRSS was not the primary outcome measure, it was captured by expert centers in the USA and assessed by the same investigator for a given patient. Second, we have validated the MCID estimates for the mRSS using patient-driven anchors; all previous estimates were based on physician anchors [21] or consensus agreement. Our effect size in this analysis for the MCID groups (range of 0.48–0.53) is very similar to the estimates provided by Khanna et al. ([5] effect sizes for the MCID group were between 0.40–0.66). Third, MCID estimates are an approximation, and experts have suggested using multiple anchors to define a range for these estimates [4] so we included three anchors in the current analysis. Fourth, our analysis provided MCID estimates that correspond to patient-reported outcome changes over time, supporting the validity of these estimates.

Our study is not without limitations. First, the analysis was post hoc rather than a priori. Second, the correlation between the anchors and the mRSS was less than the proposed cutoff point of 0.30, adding to the uncertainty of these estimates. For example, the relationship between the SF-36 health transition and PtGA anchors versus the mRSS outcome may have been influenced by changes in variables unrelated to the skin, including lungs, gastrointestinal involvement, and other disease manifestations. Further analyses of ongoing clinical trials should explore other anchors that focus on skin involvement rather than global disease and that have better associations with the mRSS.

## Conclusion

We report patient-based MCID estimates for the mRSS using data from two large randomized controlled trials. These mRSS MCID estimates can be used for interpretation of ongoing clinical trials in SSc and interstitial lung disease, and for sample size estimation in future trials.

## Abbreviations

BDI: Baseline Dyspnea Index
dcSSc: Diffuse cutaneous systemic sclerosis
HAQ-DI: Health Assessment Questionnaire- Disability Index
lcSSc: Limited cutaneous systemic sclerosis
MCID: Minimal clinically important difference
mRSS: Modified Rodnan Skin Score
PtGA: Patient Global Assessment
SF-36: Short Form-36
SLS: Scleroderma Lung Studies
SSc: Systemic sclerosis
TDI: Transition Dyspnea Index

## References

[1] Denton CP, Khanna D. Systemic sclerosis. Lancet. 2017;390(10103):1685-169. 10.1016/S0140-6736(17)30933-9 28413064

[2] Khanna D, Furst DE, Clements PJ, Allanore Y, Baron M, Czirjak L, Distler O, Foeldvari I, Kuwana M, Matucci-Cerinic M (2017). Standardization of the modified Rodnan skin score for use in clinical trials of systemic sclerosis. J Scleroderma Relat Disord.

[3] Hays RD, Woolley JM (2000). The concept of clinically meaningful difference in health-related quality-of-life research. How meaningful is it?. Pharmacoeconomics.

[4] Revicki D, Hays RD, Cella D, Sloan J (2008). Recommended methods for determining responsiveness and minimally important differences for patient-reported outcomes. J Clin Epidemiol.

[5] Khanna D, Furst DE, Hays RD, Park GS, Wong WK, Seibold JR, Mayes MD, White B, Wigley FF, Weisman M (2006). Minimally important difference in diffuse systemic sclerosis: results from the d-penicillamine study. Ann Rheum Dis.

[6] Tashkin DP, Elashoff R, Clements PJ, Goldin J, Roth MD, Furst DE, Arriola E, Silver R, Strange C, Bolster M (2006). Cyclophosphamide versus placebo in scleroderma lung disease. N Engl J Med.

[7] Tashkin DP, Roth MD, Clements PJ, Furst DE, Khanna D, Kleerup EC, Goldin J, Arriola E, Volkmann ER, Kafaja S (2016). Mycophenolate mofetil versus oral cyclophosphamide in scleroderma-related interstitial lung disease (SLS II): a randomised controlled, double-blind, parallel group trial. Lancet Respir Med.

[8] Namas R, Tashkin DP, Furst DE, Wilhalme H, Tseng CH, Roth MD, Kafaja S, Volkmann E, Clements PJ, Khanna D (2018). Efficacy of mycophenolate mofetil and oral cyclophosphamide on skin thickness: post hoc analyses from two randomized placebo-controlled trials. Arthritis Care Res.

[9] Khanna D, Yan X, Tashkin DP, Furst DE, Elashoff R, Roth MD, Silver R, Strange C, Bolster M, Seibold JR (2007). Impact of oral cyclophosphamide on health-related quality of life in patients with active scleroderma lung disease: results from the scleroderma lung study. Arthritis Rheum.

[10] Mahler DA, Weinberg DH, Wells CK, Feinstein AR (1984). The measurement of dyspnea. Contents, interobserver agreement, and physiologic correlates of two new clinical indexes. Chest.

[11] Fries JF, Spitz P, Kraines RG, Holman HR (1980). Measurement of patient outcome in arthritis. Arthritis Rheum.

[12] Clements PJ, Wong WK, Hurwitz EL, Furst DE, Mayes M, White B, Wigley F, Weisman M, Barr W, Moreland L (1999). Correlates of the disability index of the health assessment questionnaire: a measure of functional impairment in systemic sclerosis. Arthritis Rheum.

[13] Ware JE (2000). SF-36 health survey update. Spine (Phila Pa 1976).

[14] Khanna D, Furst DE, Clements PJ, Park GS, Hays RD, Yoon J, Korn JH, Merkel PA, Rothfield N, Wigley FM (2005). Responsiveness of the SF-36 and the Health Assessment Questionnaire Disability Index in a systemic sclerosis clinical trial. J Rheumatol.

[15] Wiese AB, Berrocal VJ, Furst DE, Seibold JR, Merkel PA, Mayes MD, Khanna D (2014). Correlates and responsiveness to change of measures of skin and musculoskeletal disease in early diffuse systemic sclerosis. Arthritis Care Res.

[16] Khanna D, Tseng CH, Furst DE, Clements PJ, Elashoff R, Roth M, Elashoff D, Tashkin DP, for Scleroderma Lung Study I (2009). Minimally important differences in the Mahler's Transition Dyspnoea Index in a large randomized controlled trial—results from the Scleroderma Lung Study. Rheumatology.

[17] du Bois RM, Weycker D, Albera C, Bradford WZ, Costabel U, Kartashov A, King TE, Lancaster L, Noble PW, Sahn SA (2011). Forced vital capacity in patients with idiopathic pulmonary fibrosis: test properties and minimal clinically important difference. Am J Respir Crit Care Med.

[18] Kafaja S, Clements P, Wilhalme H, Furst D, Tseng CH, Hyun K, Goldin J, Volkmann E, Roth M, Tashkin D et al: Reliability and minimal clinically important differences (MCID) of forced vital capacity: post-hoc analyses from the Scleroderma Lung Studies (SLS-I and II). The American College of Rheumatology Annual Meeting in Washington, DC (November 2016) (Oral Presentation, Abstract 971).

[19] Khanna D, Hays RD, Shreiner AB, Melmed GY, Chang L, Khanna PP, Bolus R, Whitman C, Paz SH, Hays T (2017). Responsiveness to change and minimally important differences of the Patient-Reported Outcomes Measurement Information System Gastrointestinal Symptoms scales. Dig Dis Sci.

[20] Khanna PP, Furst DE, Clements PJ, Maranian P, Indulkar L, Khanna D, Investigators DP (2010). Tendon friction rubs in early diffuse systemic sclerosis: prevalence, characteristics and longitudinal changes in a randomized controlled trial. Rheumatology.

[21] Gazi H, Pope JE, Clements P, Medsger TA, Martin RW, Merkel PA, Kahaleh B, Wollheim FA, Baron M, Csuka ME (2007). Outcome measurements in scleroderma: results from a delphi exercise. J Rheumatol.

[22] Maurer B, Graf N, Michel BA, Muller-Ladner U, Czirjak L, Denton CP, Tyndall A, Metzig C, Lanius V, Khanna D (2015). Prediction of worsening of skin fibrosis in patients with diffuse cutaneous systemic sclerosis using the EUSTAR database. Ann Rheum Dis.

[23] Fleming TR, Powers JH (2012). Biomarkers and surrogate endpoints in clinical trials. Stat Med.

